# Supplementary material for: Multivariate extremes in lakes
Source: Nat Commun. 2024 May 29;15:4559. doi: 10.1038/s41467-024-49012-7 (PMC11137041; doi:10.1038/s41467-024-49012-7)
Supplement: Supplementary file 1 — Supplementary Information [file 41467_2024_49012_MOESM1_ESM.pdf]

Supplementary Information

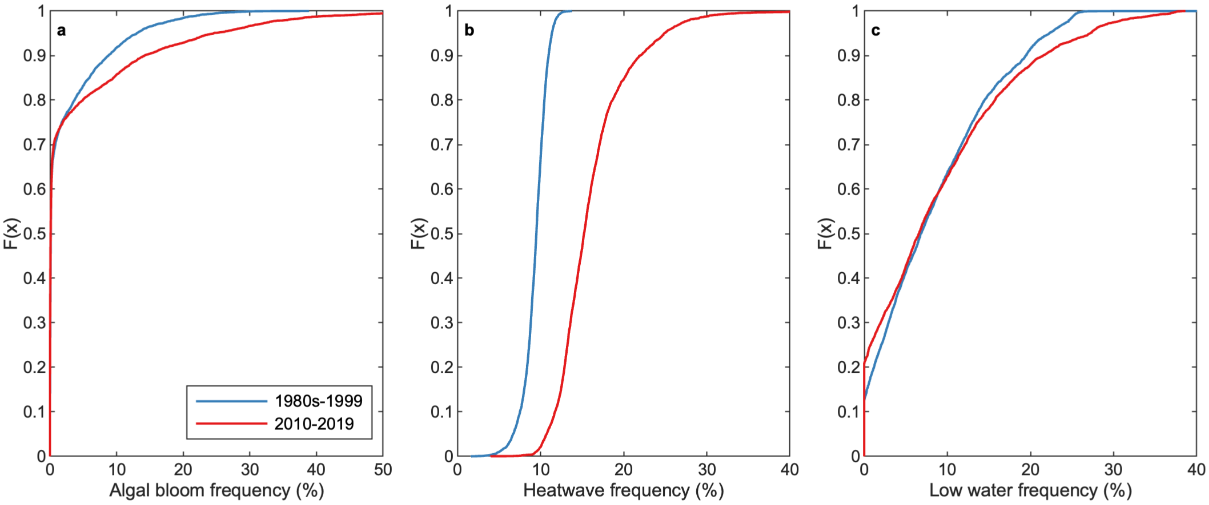

Supplementary Figure 1 | Significant change in extreme events. Empirical cumulative distribution plots between the historic (~1980s to 1999; blue) and contemporary (2010 to 2019; red) periods for the frequency of (**a**) algal blooms, (**b**) lake heatwaves, and (**c**) low water extremes in the 2,724 studied sites.

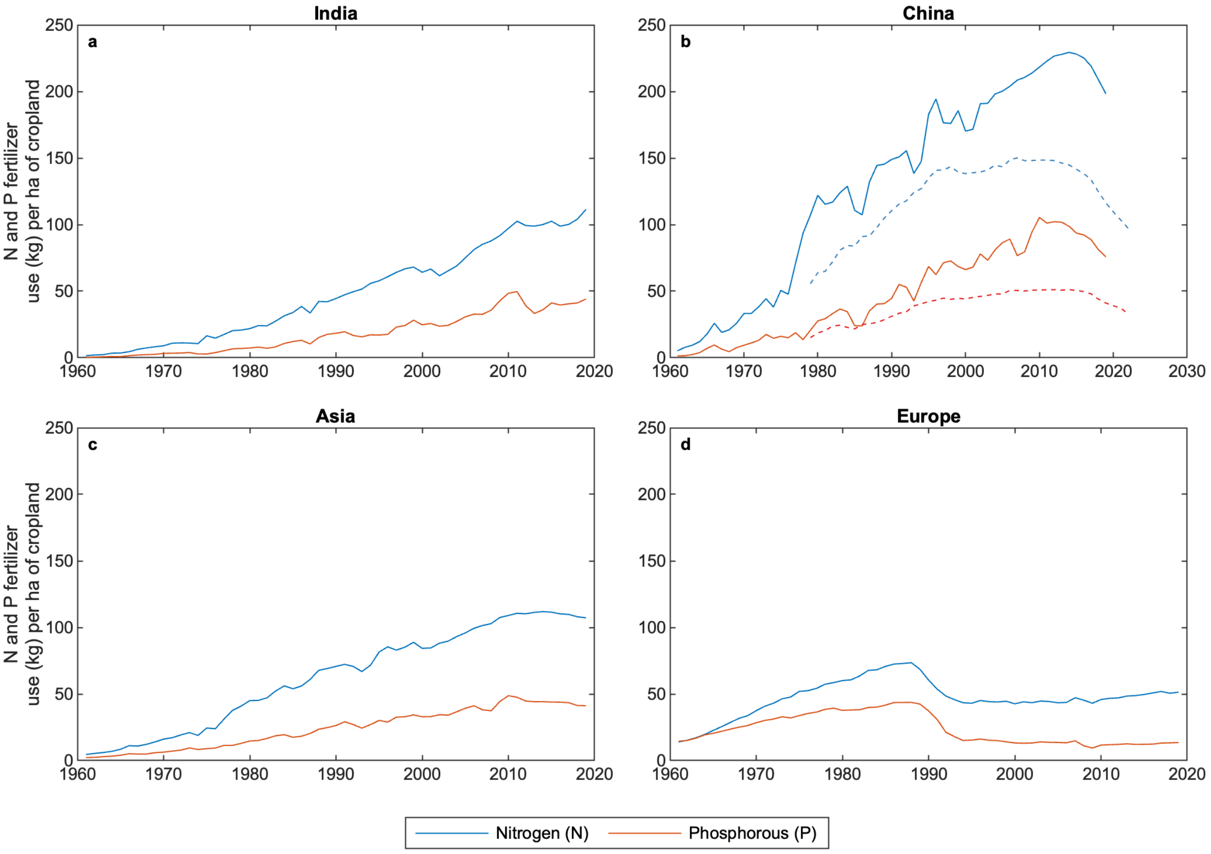

Supplementary Figure 2 | Historic changes in national- and continental-scale annual fertilizer consumption. Shown are estimated changes in (**a**) India, (**b**) China, (**c**) Asia (**d**) Europe. These data describe the estimated total amount of fertilizers (Nitrogen [N] and Phosphorus [P]) per area of cropland. Data for each example are from ref. 72. We also show in panel **b**, total N and P per hectare of cropland in China using data (dashed lines) from the National Bureau of Statistics of China (http://www.stats.gov.cn/english/).

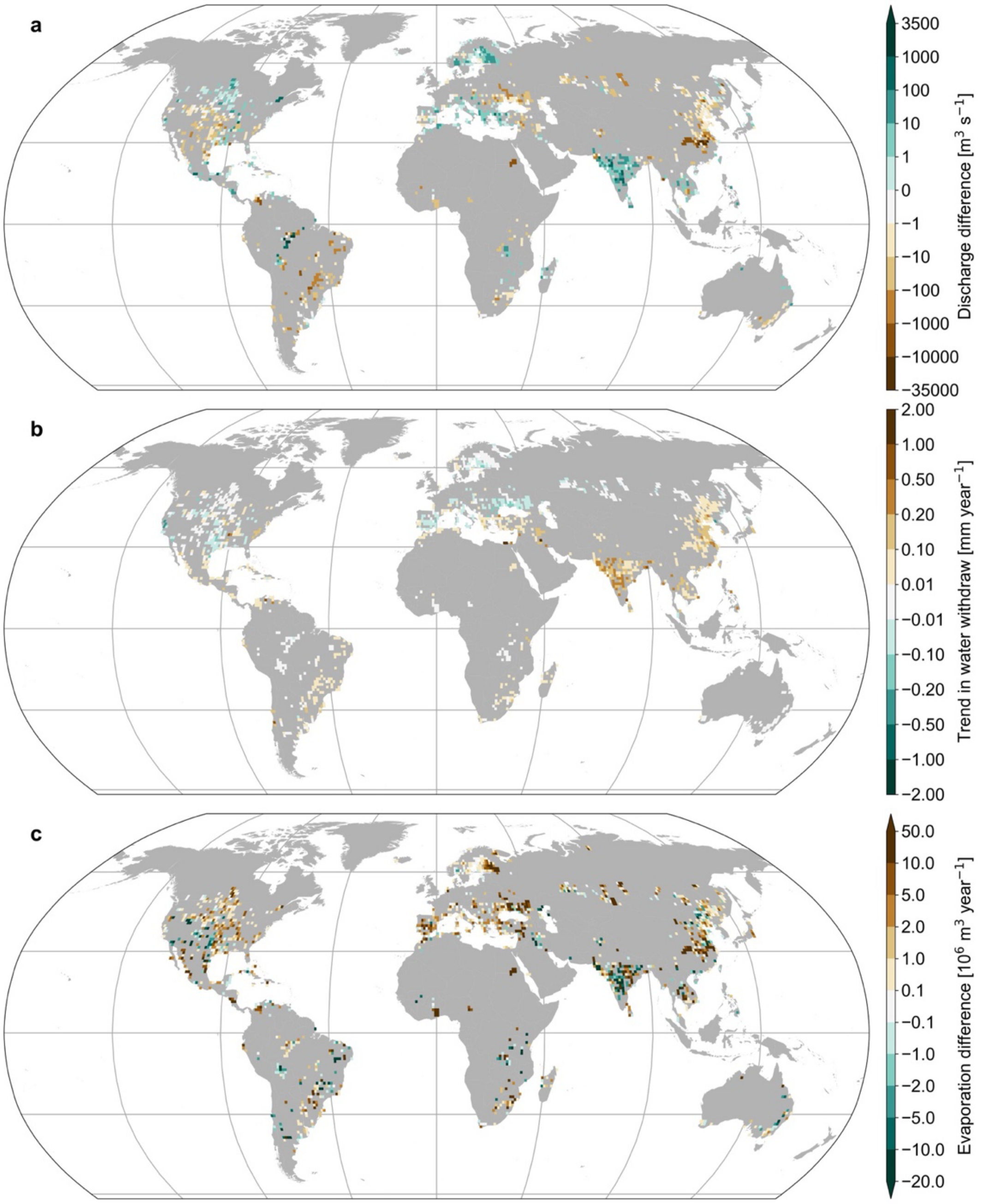

Supplementary Figure 3 | External drivers of lake level change. (a) Long-term changes in naturalized streamflow ($m^3\ s^{-1}$) between the historic (1985 to 1999) and the contemporary (2010 to 2019) period. Streamflow data were retrieved from the GloFAS-ERA5 dataset with a spatial resolution of 0.1° (ref. 22). **(b)** Long-term trend of total water (surface water and groundwater) withdrawal (mm $year^{-1}$) from 1971 to 2010. Annual water withdraw data were retrieved from the reconstructed gridded (0.5° spatial resolution) water withdraw dataset[62]. **(c)** Changes in open-water evaporation volume ($10^6\ m^3\ year^{-1}$). Data are aggregated into 1° × 1° grid cells.

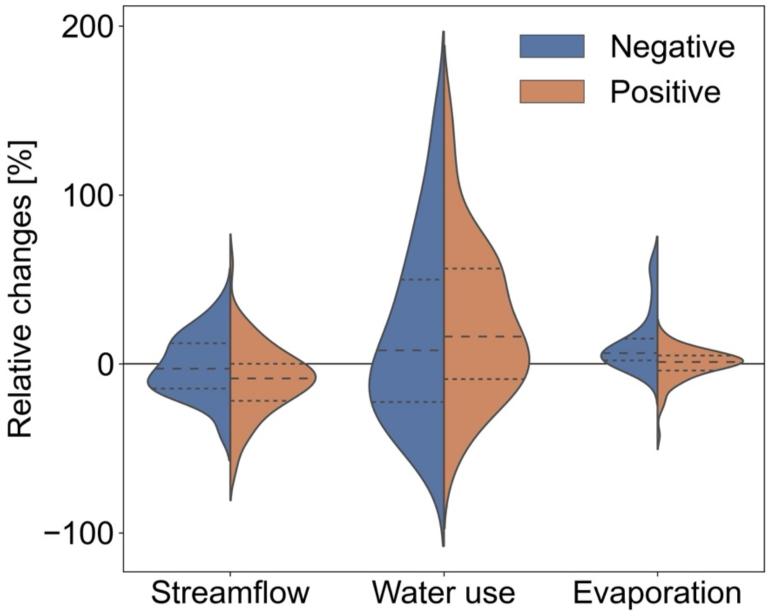

Supplementary Figure 4 | Relative change in external drivers of lake level change Influence of streamflow, water use, and open-water evaporation on the occurrence of low water extreme events between 1980s-1999 and 2010-2019. The two dominant drivers of change in low water extremes are streamflow and water use. We observe that positive changes in low water extremes are associated with larger decrease of streamflow and larger increase of water use.

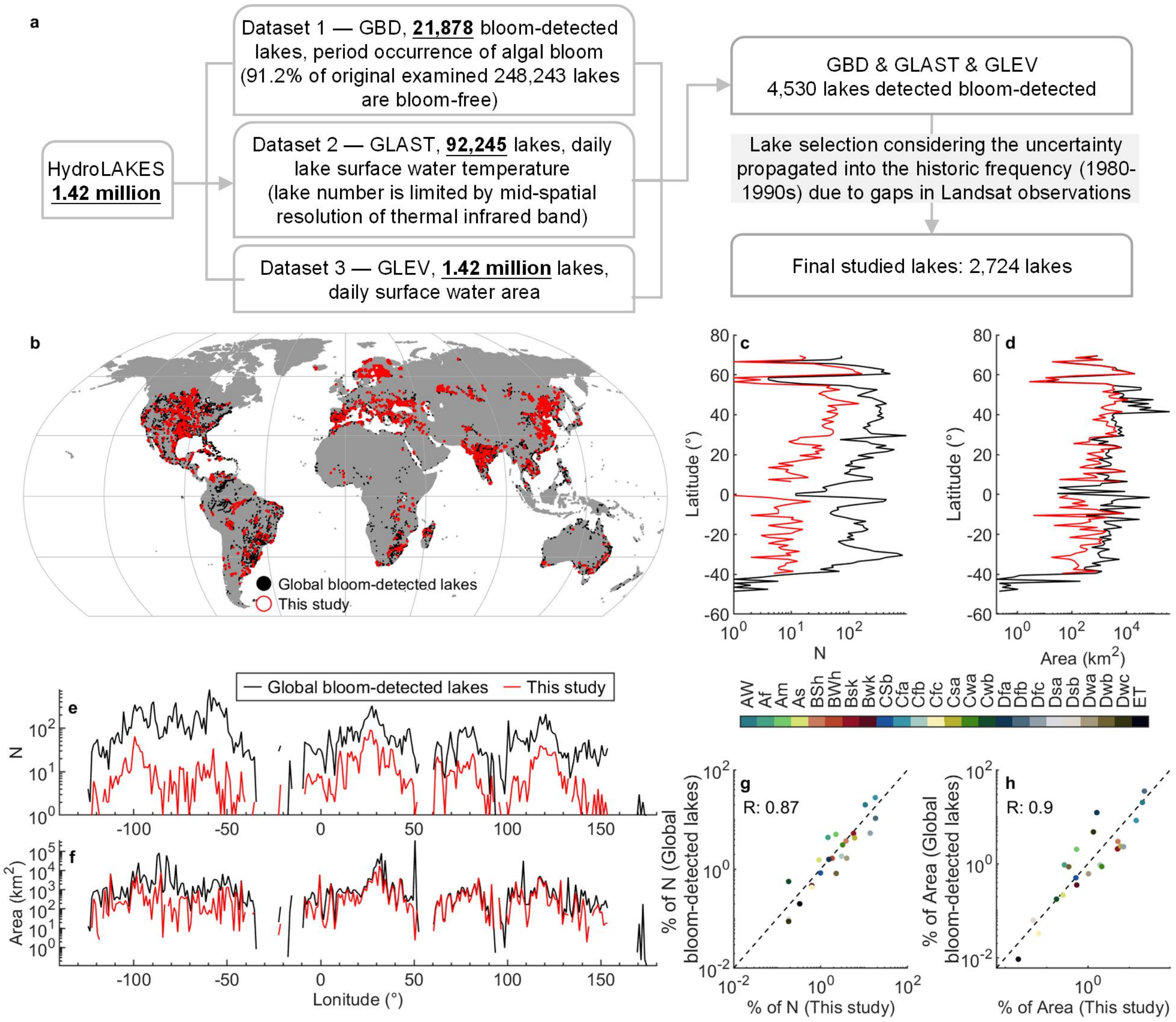

Supplementary Figure 5 | Representativeness of examined lakes compared to global bloom-affected lakes. Shown is (**a**) the determination process of the studied lakes, and (**b**) their spatial distribution as well as the (**c-d**) longitudinal profiles and (**e-f**) latitudinal profiles of lake numbers (**c, e**) and lake areas (**d, f**). In panels (**b-f**), black dots or curves represent global bloom-affected lakes, and red dots or curves represent the studied lakes. Also shown is (**g-h**) comparison of the percentage (%) of lakes (**g**, number; **h**, area) in various climatic zones between the studied lakes and global bloom-affected lakes. Climate zones are classified according to the Köppen–Geiger world map of climate classification[1] (http://koeppen-geiger.vu-wien.ac.at/present.htm). The annotations of 'A*', 'B*', 'C*', 'D*', and 'E*' in the legend of panels (**g**) and (**h**) represent tropical, arid, temperate, cold, and polar climate zones, respectively.

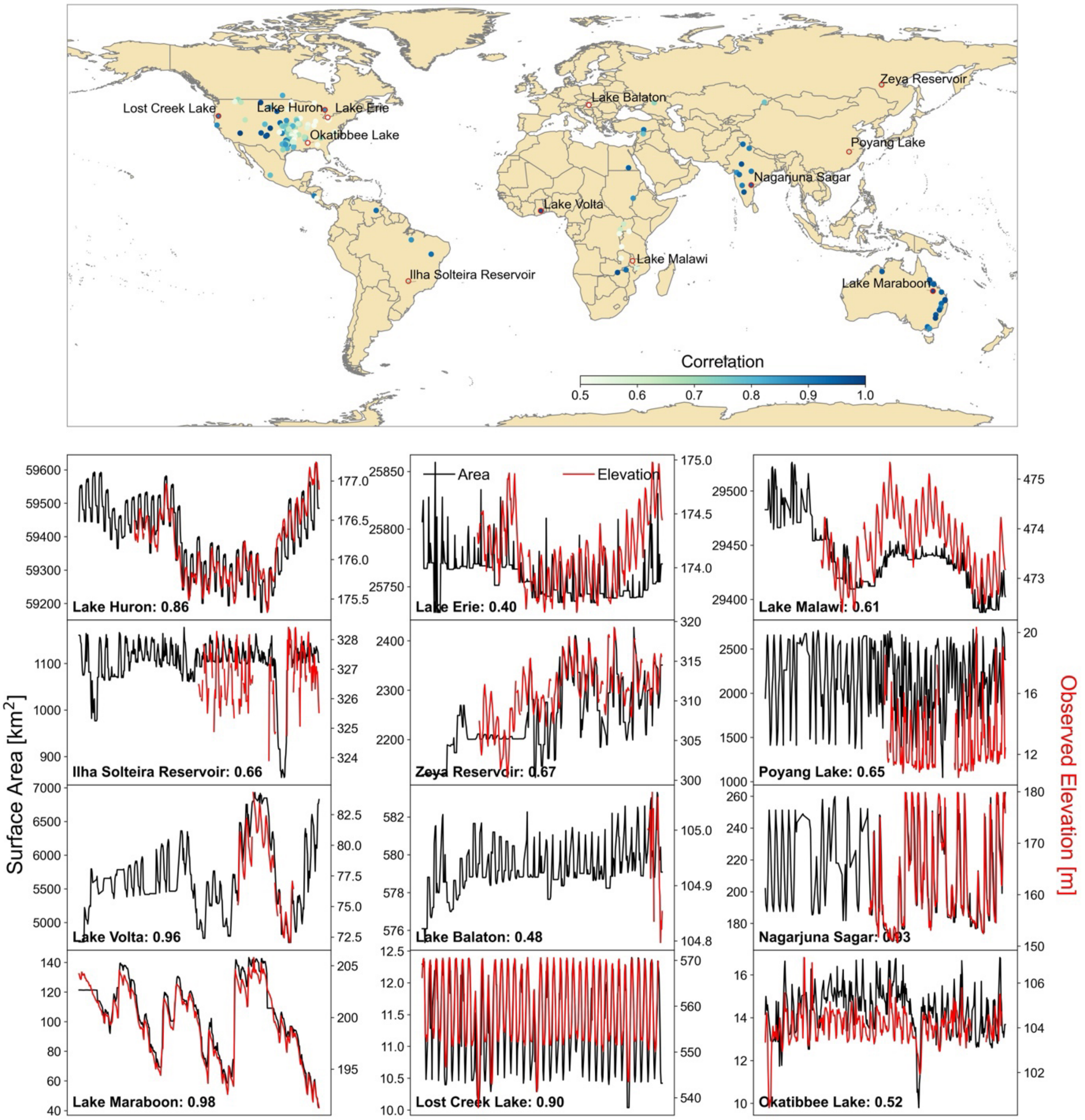

Supplementary Figure 6 | Surface area validation of satellite observations. Shown is a comparison between remotely sensed lake surface area and observed lake elevation for 155 globally distributed lakes. The observed lake elevation data were collected from multiple sources including Bureau of Meteorology in Australia, Central Water Commission in India, US Army Corps of Engineers, and DAHITI. Detailed information on these lakes and their correlation coefficients can be found in Supplementary Table 7. The median correlation coefficient is 0.76. Also shown are time series of satellite-derived (black) and observed elevation data (red) from representative lakes across all continents. Twelve lakes were randomly selected for all continents to show the monthly time series for both surface area and observed elevation.

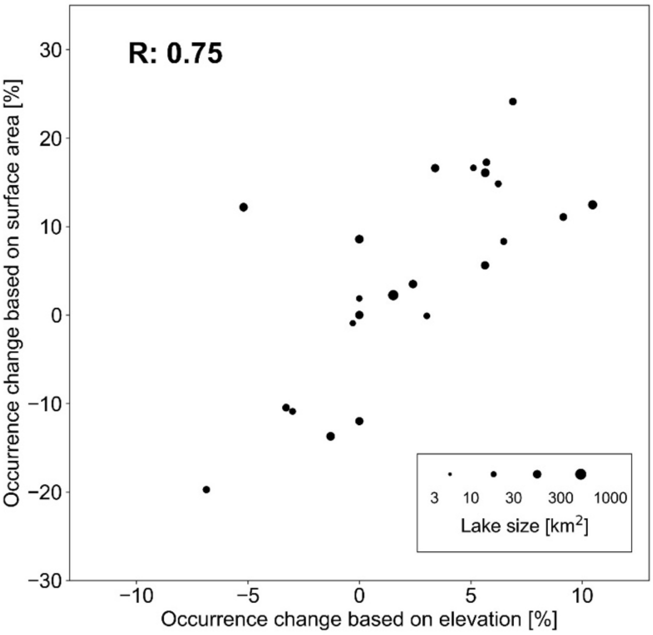

Supplementary Figure 7 | Validation of lake surface area change. Validation of area-based lake extreme occurrences using elevation-based calculations for 25 lakes. These 25 lakes were selected from the 155 lakes in Supplementary Table 7 that have complete data records from 1985 to 2019.

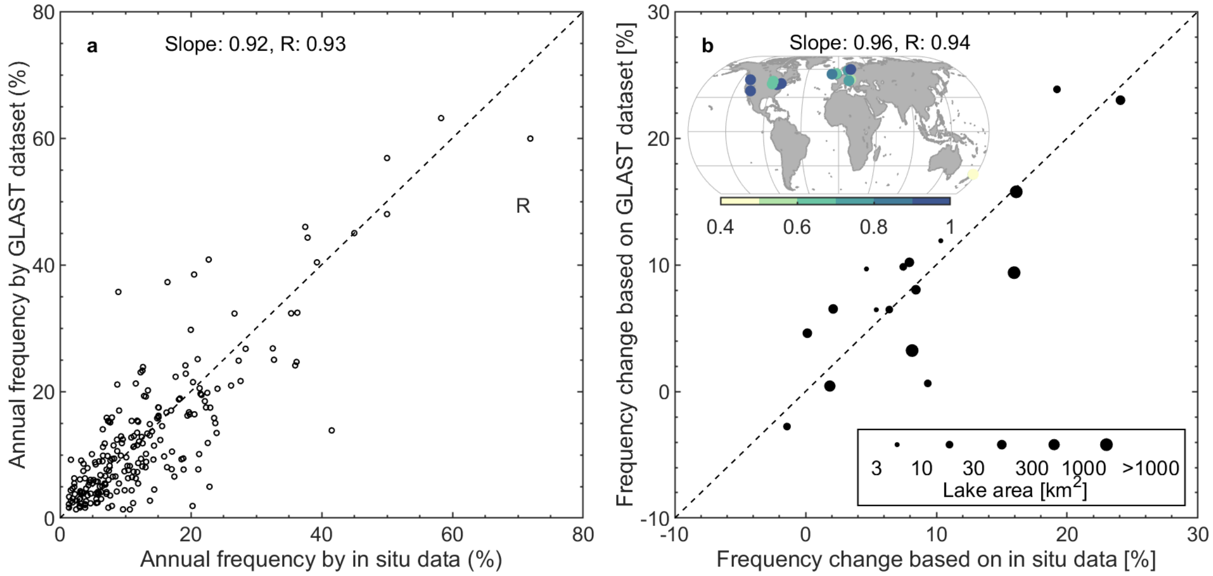

Supplementary Figure 8 | Lake heatwave validation. Validation of GLAST-based lake heatwave frequencies using in situ data-based calculations for 17 lakes that have continuous in situ daily water surface temperature records. Shown are comparisons between GLAST-based and in situ temperature based annual frequency results **(a)** and frequency change **(b)**. The map in panel **(b)** presents the distribution of in situ lakes, color-coded based on the correlation coefficient between GLAST calculated and in situ data derived annual heatwave frequency.

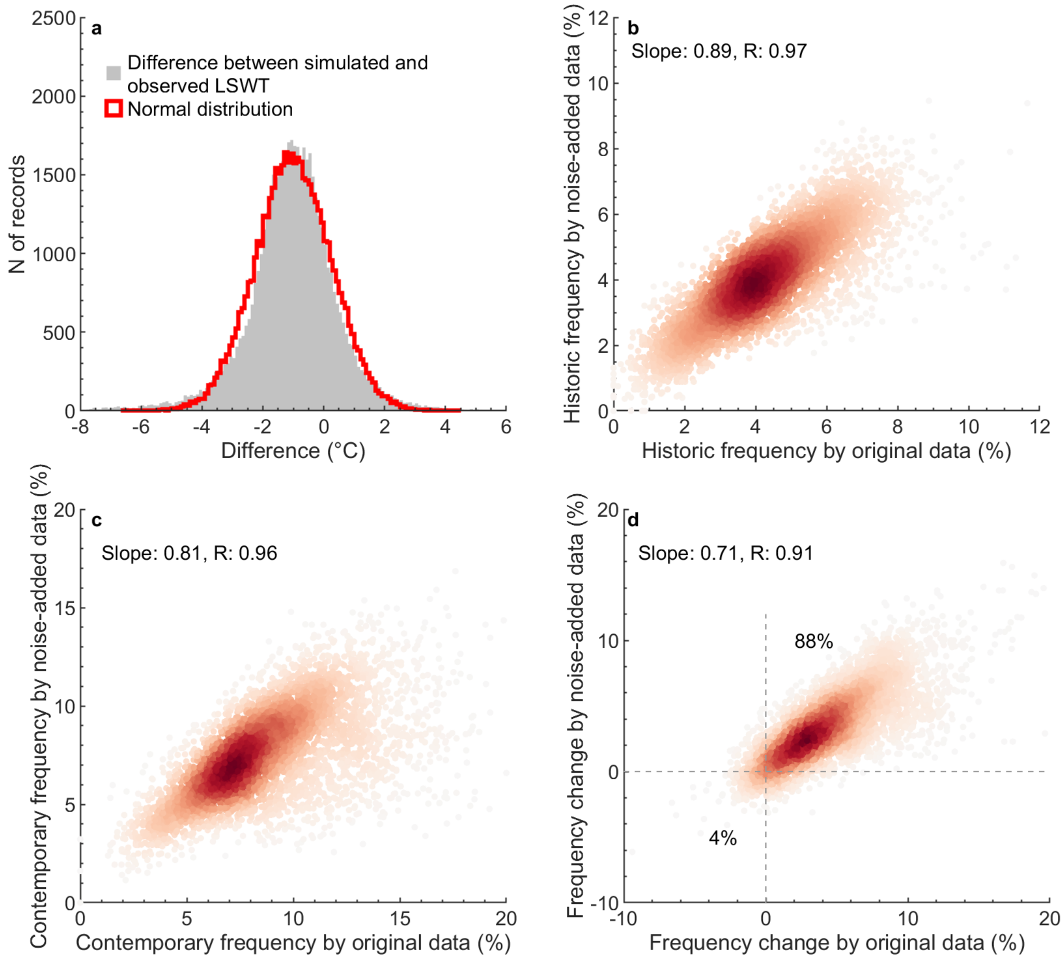

Supplementary Figure 9 | Uncertainty of change in lake heatwave frequency. Quantification of the robustness of the lake heatwave frequency between the two focused periods (i.e., historic, ~1980s-1999 and contemporary, 2010s) influenced by the uncertainty of the daily GLAST dataset. **(a)** The grey histogram illustrates differences between the FLake daily simulations and in situ dataset. The red histogram represents the generated random noises following the same distribution as the differences observed in the grey histogram. **(b-d)** Density scatterplots of lake heatwave frequency in the historic period **(b)** and contemporary period **(c)** as well as the frequency change from the historic to contemporary period **(d)** between the noise-added and original data for the examined lakes. The linear slopes and correlation coefficients (R) are annotated in each panel.

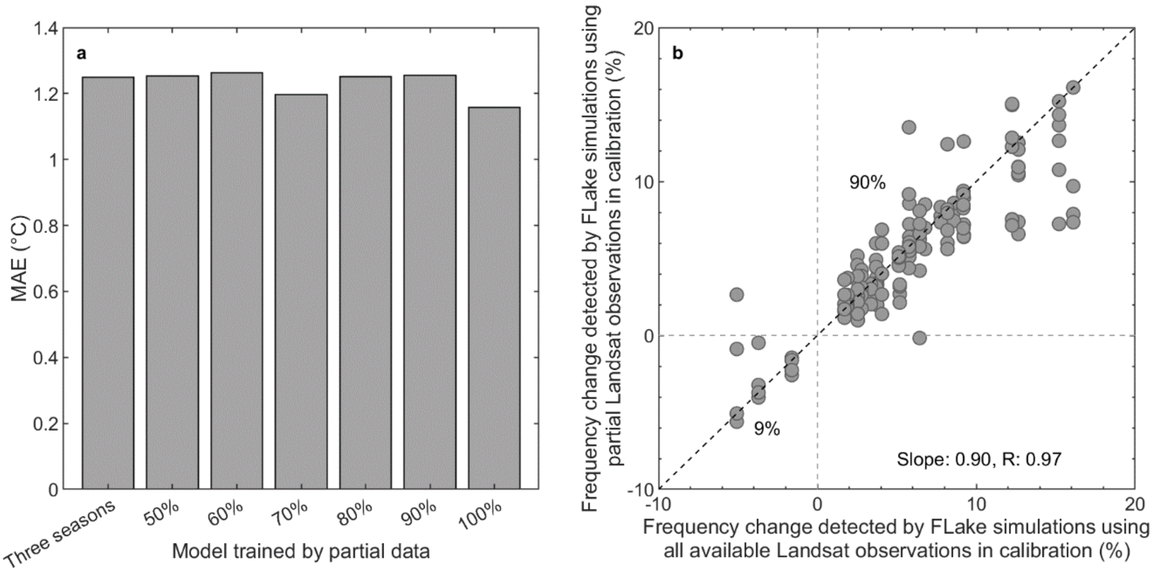

Supplementary Figure 10 | The influence of Landsat observation quantity on FLake simulations and heatwave detections. Shown is **(a)** comparison of validation performance of FLake simulations using in situ dataset, with FLake models calibrated using different percentages of available Landsat observations. Also shown is **(b)** comparison of heatwave frequency change detected by FLake simulations calibrated using total available Landsat observations and using a specific proportion of the original data.

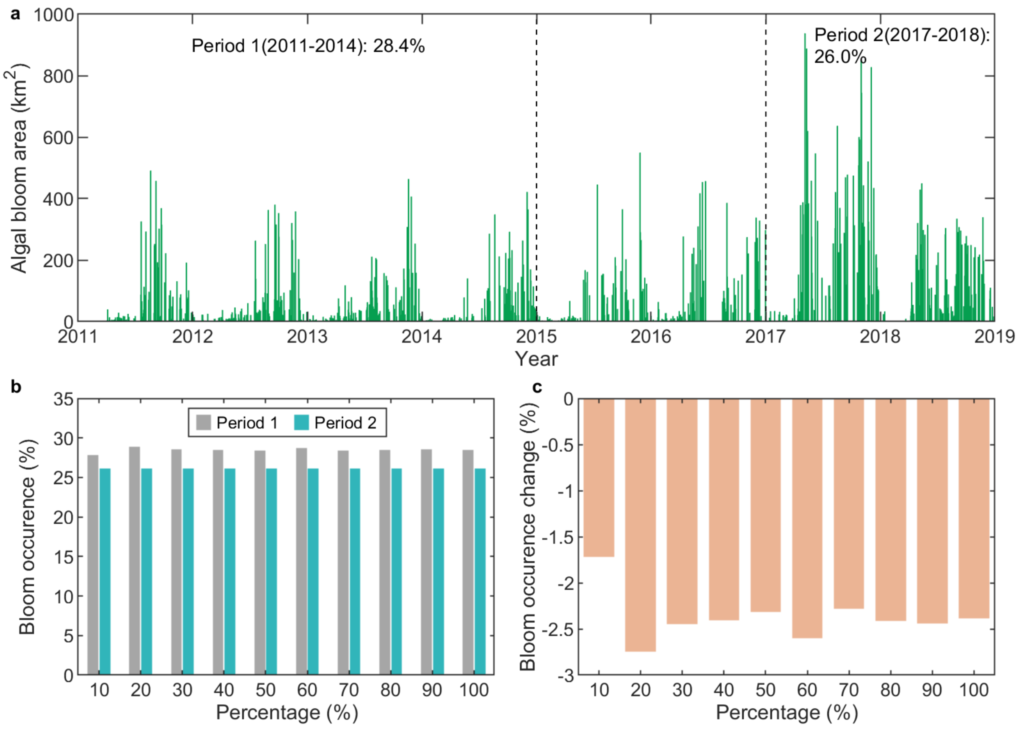

Supplementary Figure 11 | Algal bloom frequency comparison. Comparison of algal bloom frequency changes during two periods under different number of observation data in Lake Taihu. **(a)** Daily algal bloom areas from 2011 to 2018 extracted from GOCI data for Lake Taihu[71]. Algal bloom frequencies for two periods are calculated: the first period represents the first half of the entire data period (i.e., 2011-2014; period 1), and the second period represents the last two years (2017-2018; period 2). **(b)** Comparison of algal bloom frequencies during period 1 under different percentage levels of observations, indicated in grey, and algal bloom frequency during period 2 (marked as 26% in subfigure a) when observations are at 100% (i.e., higher observation quantities). **(c)** Changes in algal bloom frequencies for both periods concerning variations in observation quantities during period 1. Consistent negative values indicate that changes in Lake Taihu's algal bloom frequency are not influenced by inconsistencies in observation quantities between the two periods.

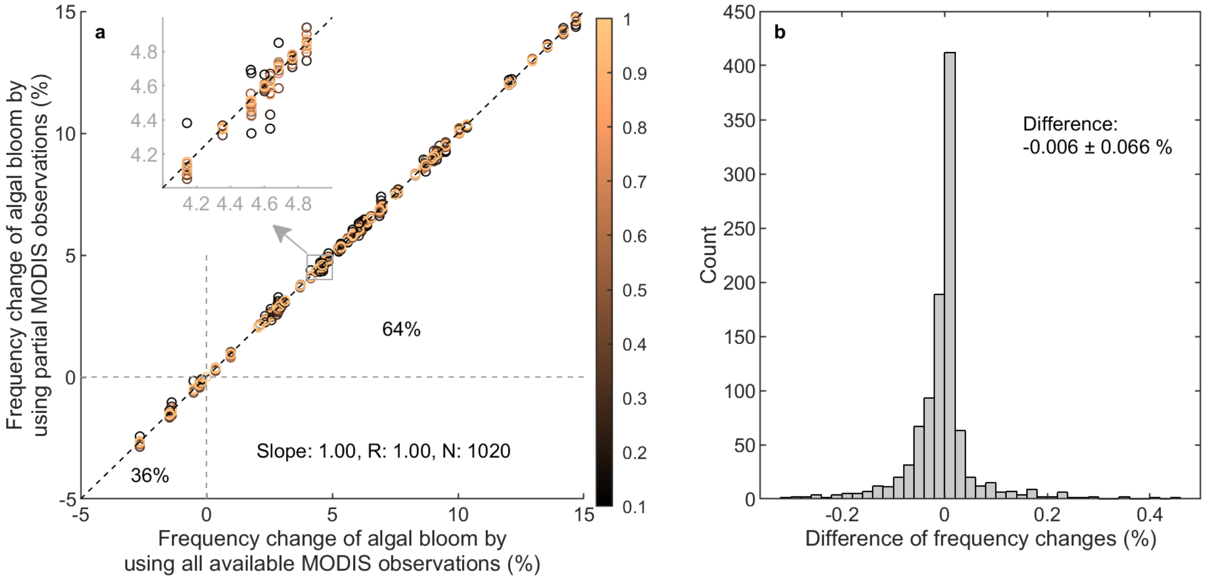

Supplementary Figure 12 | MODIS-derived information on algal bloom frequency. The influence of MODIS observation quantity on algal bloom frequency change during the historic period of 2003-2012 and present period of 2018-2022. Shown is (**a**) comparison of algal bloom frequency change detected using all available MODIS observations and using a specific proportion of the original data for historical period frequency calculations. Different colors represent varying percentage of observations used. A zoomed-in panel highlights the details of their differences. Also shown is (**b**) the histogram of the difference between the two frequency changes, with mean (-0.006%) and standard deviation (i.e., uncertainty, 0.066%) annotated. Frequency is calculated based on daily algal bloom area data for 102 lakes in China from 2003-2022 (ref. 9). For each lake, 10 choices of proportion were conducted.

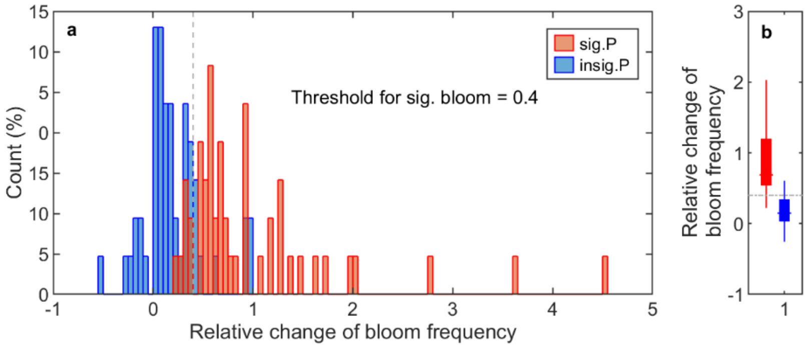

Supplementary Figure 13 | Statistical significance of trends in algal bloom frequency. Comparison of relative changes in lake algal bloom frequency between significantly and insignificantly changing long-term trends. Shown are the (**a**) histogram and (**b**) boxplot depicting the comparison between the two groups.

Supplementary Table 1 | National-scale summaries of the calculated change (mean and standard deviation [SD]) in the occurrence frequency (%) of algal blooms, low water extremes and lake heatwaves, as well as the average (and standard deviation) change in lake surface water temperature (LSWT) and lake water extent (LWE).

Country	Bloom Occurrence (mean)	Bloom Occurrence (SD)	Heatwaves (mean)	Heatwaves (SD)	Low water extreme (mean)	Low water extreme (SD)	LSWT (mean)	LSWT (SD)	LWE (mean)	LWE (SD)	Number of lakes
Afghanistan	11.91	NA	5.15	NA	-19.67	NA	0.41	NA	4.09	NA	1
Albania	1.78	3.72	9.89	1.70	-1.73	15.96	0.96	0.03	3.25	8.29	7
Algeria	0.12	0.22	4.57	2.78	-4.66	13.66	0.39	0.08	10.14	19.56	8
Argentina	-0.06	0.12	0.91	1.04	2.75	20.11	0.20	0.11	-1.65	21.80	42
Australia	-0.03	0.12	2.83	1.90	-0.30	13.16	0.49	0.18	-0.54	16.57	32
Bangladesh	11.98	14.27	2.64	1.76	-12.33	11.29	0.23	0.03	26.64	37.17	7
Belize	0.23	0.16	4.58	1.57	-3.55	10.45	0.24	0.01	-7.08	3.90	3
Bolivia	-0.10	0.09	2.25	0.89	5.09	18.21	0.24	0.05	0.32	13.06	34
Bosnia and Herzegovina	-1.65	3.08	11.92	2.46	13.83	16.19	0.68	0.53	-3.67	3.11	2
Botswana	-0.04	NA	6.73	NA	37.46	NA	0.26	NA	-29.30	NA	1
Brazil	0.01	0.09	5.59	3.92	2.36	18.15	0.33	0.13	10.99	57.90	136
Bulgaria	3.32	3.60	8.51	2.64	-9.48	19.69	0.93	0.07	10.23	15.65	5
Burkina Faso	-0.02	NA	5.86	NA	-3.84	NA	0.38	NA	-5.47	NA	1
Burundi	-0.11	NA	8.73	NA	-25.92	NA	0.29	NA	5.59	NA	1
Cambodia	6.07	16.16	7.12	1.82	2.21	13.94	0.26	0.08	0.49	15.94	6
Cameroon	0.28	NA	9.27	NA	-2.04	NA	0.46	NA	4.35	NA	1
Canada	0.02	0.07	-0.24	1.83	-5.51	7.51	0.17	0.13	4.22	9.36	85
Chile	0.08	0.17	-2.14	5.29	12.42	21.52	-0.08	0.13	16.30	76.31	5
China	7.12	9.18	1.53	1.87	0.50	12.09	0.33	0.16	3.28	22.72	404
Colombia	0.06	0.12	9.53	1.53	0.75	16.46	0.45	0.06	14.71	35.05	32
Cuba	0.09	0.20	4.66	2.00	4.76	18.78	0.12	0.05	6.98	18.58	8
Czech Republic	0.24	NA	7.72	NA	-15.46	NA	0.37	NA	18.12	NA	1
Democratic Republic of the Congo	0.04	0.04	5.50	3.49	13.63	14.45	0.25	0.12	-8.66	7.44	5
Denmark	-5.39	NA	2.89	NA	-20.74	NA	0.62	NA	3.26	NA	1
Dominican Republic	-0.09	0.20	6.35	2.10	2.43	13.81	0.25	0.03	7.63	12.12	5
Egypt	0.14	0.19	8.30	1.06	-2.83	18.32	0.68	0.13	5.66	10.86	4
El Salvador	0.25	NA	4.21	NA	-2.10	NA	0.07	NA	-0.14	NA	1
Estonia	-0.28	NA	5.54	NA	13.65	NA	0.17	NA	-4.85	NA	1

Finland	-0.40	2.85	8.48	2.92	3.19	7.21	0.40	0.25	-0.70	1.72	365
France	0.32	6.18	5.63	2.84	-4.44	15.98	0.54	0.13	1.83	6.14	20
Georgia	-1.11	1.62	17.88	6.91	0.11	15.35	0.98	0.08	2.04	2.01	3
Germany	0.08	NA	6.56	NA	21.48	NA	0.64	NA	-7.03	NA	1
Ghana	0.03	0.07	7.87	3.40	14.27	2.25	0.34	0.11	-2.05	6.55	3
Greece	3.20	4.98	8.34	3.72	-13.25	11.73	0.79	0.11	7.62	9.32	18
Haiti	-0.02	NA	4.88	NA	0.35	NA	0.25	NA	1.33	NA	1
Hungary	0.05	4.69	8.17	1.23	-11.45	10.60	0.58	0.18	12.48	27.72	6
Iceland	2.54	2.86	5.81	1.87	6.31	6.90	0.34	0.17	0.13	1.06	6
India	16.14	13.98	4.33	1.87	1.30	14.63	0.28	0.10	39.67	216.25	145
Iran	14.63	0.62	7.62	6.18	8.11	15.68	0.53	0.22	-14.29	25.53	2
Iraq	11.31	13.63	9.09	3.58	6.12	16.68	0.83	0.06	-7.77	28.48	10
Italy	6.43	10.29	7.83	2.74	-9.47	13.75	0.67	0.16	15.22	22.37	26
Jamaica	0.12	NA	-0.03	NA	2.50	NA	0.06	NA	-0.33	NA	1
Japan	1.10	2.71	6.47	1.12	3.25	9.67	0.44	0.15	-1.12	1.69	10
Kazakhstan	3.65	5.32	2.07	1.59	3.69	11.57	0.30	0.19	-0.51	21.90	75
Kenya	0.09	0.07	7.32	0.42	-5.77	10.79	0.32	0.06	1.85	13.26	2
Laos	4.04	2.31	4.23	0.98	-19.95	0.34	0.17	0.02	26.69	8.38	2
Latvia	-0.04	0.01	5.42	0.60	7.66	0.51	0.20	0.01	-7.09	6.91	2
Macedonia	3.55	NA	14.76	NA	12.39	NA	0.91	NA	-0.76	NA	1
Madagascar	0.00	0.14	10.77	1.97	-3.82	17.82	0.43	0.07	2.67	11.17	12
Malaysia	-1.87	NA	7.20	NA	-19.27	NA	0.21	NA	4.61	NA	1
Mali	0.17	0.05	4.97	0.41	2.03	7.17	0.39	0.06	-4.44	1.04	2
Mexico	0.06	0.13	4.17	3.52	-3.45	14.41	0.23	0.13	10.47	25.84	60
Moldova	7.62	9.54	7.13	1.80	15.39	16.10	0.44	0.10	-11.31	16.06	5
Mongolia	0.80	4.16	2.60	2.37	6.03	9.26	0.64	0.20	-13.23	13.03	6
Montenegro	2.72	NA	9.24	NA	-4.98	NA	0.92	NA	1.44	NA	1
Morocco	0.05	0.13	3.86	1.72	-5.05	15.60	0.26	0.06	13.17	25.04	9
Mozambique	0.10	0.20	2.50	2.52	-10.74	21.21	0.17	0.15	22.66	40.18	10
Myanmar	3.41	7.95	3.96	2.40	-7.27	16.33	0.27	0.11	1145.48	1971.04	3
Netherlands	5.23	NA	4.74	NA	-11.13	NA	0.44	NA	-0.32	NA	1
Nicaragua	0.11	0.29	4.53	2.42	-5.28	21.61	0.23	0.04	4.28	24.90	5
North Korea	5.24	11.52	3.67	1.85	14.29	5.10	0.53	0.12	-4.03	4.62	4
Norway	-0.43	1.22	4.25	3.18	-0.41	8.11	0.43	0.19	0.30	1.99	62
Pakistan	3.61	13.61	4.53	2.02	-8.57	16.25	0.50	0.06	9.74	20.93	18
Paraguay	-0.01	0.06	0.25	1.06	-7.10	11.73	0.30	0.07	1.43	2.99	3
Peru	0.19	0.26	1.17	3.30	-11.43	14.05	0.27	0.09	13.53	21.36	6
Philippines	4.49	13.06	4.85	0.22	17.39	15.99	0.20	0.06	-2.52	26.94	2
Poland	3.35	4.58	5.46	2.30	6.75	11.53	0.87	0.23	-3.95	8.10	2
Portugal	1.98	4.37	1.91	1.51	0.47	21.31	0.31	0.06	2.94	11.57	6

Republic of Serbia	0.60	2.76	9.95	3.04	-2.46	19.14	0.44	0.15	4.18	7.00	3
Romania	3.73	7.25	8.14	2.21	3.74	15.19	0.60	0.14	4.42	27.43	22
Russia	1.92	7.70	5.02	3.84	2.98	11.36	0.42	0.28	-0.75	14.15	275
Saudi Arabia	-0.19	5.08	4.69	0.43	9.37	5.47	0.54	0.08	-1.70	1.66	2
Slovakia	2.00	2.58	7.24	1.69	13.85	19.41	0.59	0.08	-0.76	0.33	2
Slovenia	-0.04	NA	9.88	NA	7.74	NA	0.53	NA	-0.18	NA	1
South Africa	0.04	0.13	3.24	2.01	-7.79	14.61	0.16	0.09	6.59	10.55	17
South Korea	7.95	NA	6.42	NA	3.77	NA	0.88	NA	-10.28	NA	1
Spain	3.10	9.77	4.31	2.43	-10.16	15.06	0.40	0.07	35.70	48.12	41
Sri Lanka	7.25	12.04	3.12	1.38	3.38	12.81	0.21	0.08	7.82	13.56	11
Swaziland	0.05	NA	1.27	NA	5.16	NA	0.16	NA	1.03	NA	1
Sweden	-1.20	2.12	5.01	2.80	2.25	6.91	0.42	0.23	-0.33	2.59	101
Switzerland	0.00	0.03	10.09	4.05	-2.14	32.77	0.72	0.23	0.80	1.46	2
Syria	13.54	15.68	8.88	2.17	-16.37	7.54	0.75	0.05	114.08	212.76	4
Taiwan	5.23	NA	3.30	NA	15.26	NA	0.29	NA	-7.42	NA	1
Thailand	3.56	7.47	5.37	1.76	7.71	16.73	0.23	0.07	31.03	135.66	14
The Bahamas	0.30	0.11	4.93	1.23	-8.81	6.94	0.33	0.07	3.16	1.79	4
Tunisia	0.12	0.12	3.19	1.70	17.65	11.80	0.35	0.06	-3.39	12.17	5
Turkey	7.12	13.32	9.34	3.90	-1.02	15.05	0.78	0.23	21.96	82.76	44
Uganda	0.02	NA	8.96	NA	-20.50	NA	0.40	NA	17.41	NA	1
Ukraine	4.26	10.99	5.36	1.85	2.40	16.93	0.25	0.23	-0.12	13.65	52
United Kingdom	0.01	0.20	2.96	1.40	-11.58	9.67	0.32	0.14	0.60	0.33	2
United Republic of Tanzania	-0.03	0.06	8.23	1.88	7.26	17.19	0.33	0.06	-11.40	30.12	6
United States of America	0.03	0.09	3.90	2.81	0.11	15.48	0.28	0.20	2.31	20.13	328
Uruguay	-0.08	0.07	1.94	0.41	2.60	2.76	0.29	0.03	0.23	3.25	4
Venezuela	0.07	0.12	8.75	3.31	13.10	15.87	0.33	0.10	-0.61	17.51	8
Vietnam	2.37	12.37	6.09	1.27	-8.51	20.27	0.26	0.03	95.44	209.02	6
Zambia	0.03	0.08	0.61	1.45	-4.61	9.36	0.15	0.04	-0.16	2.22	6
Zimbabwe	0.15	0.27	7.18	2.03	-22.13	3.04	0.34	0.04	54.51	60.01	4

Supplementary Table 2 | National-scale summaries of the average change in fertilizer use.

**Country**	**Change in Nitrogen**	**Change in Phosphorus**
Afghanistan	2.22328574	-0.196
Albania	-5.0657508	15.6696254
Algeria	0.70050009	0.03612491
Argentina	16.1407496	12.1293754
Australia	12.0283749	-14.93175
Bangladesh	67.2621241	46.7127497
Belize	84.7689992	53.9273751
Bolivia	2.99437507	1.16125003
Bosnia and Herzegovina	65.0719995	2.73400002
Botswana	44.0889997	3.00099998
Brazil	44.5482497	42.2859993
Bulgaria	14.5406252	-12.234625
Burkina Faso	4.92912509	1.04962508
Burundi	3.00225005	2.48700003
Cambodia	16.178	8.82900002
Cameroon	2.38612499	0.44162498
Canada	32.4821265	8.44587531
Chile	91.565499	31.4984989
China	68.8399986	44.4521241
Colombia	47.7881259	27.0087497
Cuba	-38.485375	-8.1837499
Czech Republic	63.4332888	3.95571443
Democratic Republic of the Congo	0.47000001	-0.1046667
Denmark	-49.234001	-15.352875
Dominican Republic	17.4052507	5.92812517
Egypt	35.5671286	6.0121254
El Salvador	-3.576001	-2.9406247
Estonia	27.2099997	4.15750014
Finland	-19.382125	-34.033375
France	-17.101375	-42.858749
Georgia	1.60600014	3.36528575
Germany	-27.548624	-32.800375
Ghana	7.24449997	5.99524995
Greece	-40.225499	-21.577375
Haiti	NA	NA
Hungary	3.12574944	-12.567125
Iceland	-5.2826254	0.09350004

India	52.236626	24.0157502
Iran	0.49550054	-14.96325
Iraq	-7.00275	-7.1164999
Italy	-16.500375	-34.46975
Jamaica	-17.72525	-6.8998752
Japan	-27.627499	-51.853875
Kazakhstan	0.72474996	-1.5215
Kenya	10.1289998	9.86237509
Laos	NA	NA
Latvia	33.9362495	10.0915
Macedonia	NA	NA
Madagascar	3.28612493	0.26175
Malaysia	27.8492505	-5.4068747
Mali	8.86737482	1.26775
Mexico	-0.278875	6.62425014
Moldova	0.40474999	-4.9404998
Mongolia	13.5155001	-1.7946363
Montenegro	NA	NA
Morocco	7.11587492	9.03962494
Mozambique	3.65887499	0.40033334
Myanmar	5.20737507	1.45212496
Netherlands	-152.27487	-48.998
Nicaragua	10.5517501	2.77499998
North Korea	NA	NA
Norway	-1.4184988	-17.6295
Pakistan	52.135374	17.1443752
Paraguay	22.0181252	36.9543751
Peru	21.4806253	8.41812513
Philippines	16.732874	3.89299986
Poland	26.823	-3.6798751
Portugal	14.180375	-3.5048751
Republic of Serbia	NA	NA
Romania	-9.2199997	-9.3488755
Russia	1.05849986	-0.03075
Saudi Arabia	-11.825374	-22.113375
Slovakia	39.1540004	4.96828526
Slovenia	-50.429001	-41.54475
South Africa	3.01837471	-2.1406252
South Korea	-95.449752	-18.816376
Spain	4.94225059	-3.1170002
Sri Lanka	8.06337547	2.28275025
Swaziland	NA	NA

Sweden	-7.9648748	-10.512
Switzerland	-40.904249	-43.48225
Syria	-25.076875	-16.3555
Taiwan	NA	NA
Thailand	42.8136253	6.17937496
The Bahamas	79.8725818	10.2115007
Tunisia	5.53912509	1.251125
Turkey	24.0383749	4.42400012
Uganda	0.77425	0.405
Ukraine	14.3759998	2.92475004
United Kingdom	-50.143374	-29.836875
United Republic of Tanzania	3.65587498	1.6495
United States of America	17.8050007	3.78737543
Uruguay	54.3356248	33.1936247
Venezuela	17.3145001	2.16575018
Vietnam	36.6466245	42.4858749
Zambia	23.0160004	2.642375
Zimbabwe	-14.251375	-4.2989999

Supplementary Table 3 | Continental-scale summaries of the average change in fertilizer use.

**Continent**	**Change in Nitrogen**	**Change in Phosphorus**
Africa	3.50	0.54
Asia	38.72	18.30
Europe	-7.09	-15.85
North America	20.39	4.63
Oceania	14.30	-10.91
South America	31.06	26.05

Supplementary Table 4 | Continental-scale summaries of the calculated change (mean and standard deviation [SD]) in the occurrence frequency (%) of algal blooms, low water extremes and lake heatwaves, as well as the average (and standard deviation) change in lake surface water temperature (LSWT) and lake water extent (LWE).

Continent	Bloom Occurrence (mean)	Bloom Occurrence (SD)	Heatwaves (mean)	Heatwaves (SD)	Low water extreme (mean)	Low water extreme (SD)	LSWT (mean)	LSWT (SD)	LWE (mean)	LWE (SD)
Africa	0.06	0.15	5.33	3.57	-2.73	17.17	0.30	0.15	6.70	24.86
Asia	8.20	11.19	3.13	3.17	0.77	13.32	0.36	0.21	16.86	159.07
Europe	0.97	6.14	6.50	3.58	1.39	11.37	0.43	0.26	1.69	15.72
North America	0.03	0.11	3.28	3.16	-1.32	14.42	0.25	0.19	3.70	19.43
Oceania	-0.03	0.12	2.83	1.90	-0.30	13.16	0.49	0.18	-0.54	16.57
South America	-0.01	0.12	4.65	4.16	2.67	18.14	0.30	0.14	7.67	45.36

Supplementary Table 5 | Percentage of studied lakes within a country that experienced an increase in the occurrence frequency of bivariate and multivariate extremes.

	**Bivariate**			**Multivariate**
**Country**	**Change in Bloom Occurrence and Low Water**	**Change in Bloom Occurrence and Heatwave**	**Change in Heatwave and Low Water**	**Change in all three extremes**
Russia	28	47.27	52.36	27.27
Brazil	30.15	44.85	47.06	29.41
Canada	9.41	17.65	8.24	4.71
Peru	0	33.33	16.67	0
Bolivia	5.88	8.82	58.82	5.88
United States of America	22.26	59.45	38.41	21.65
Argentina	11.9	14.29	38.1	7.14
Paraguay	0	33.33	0	0
Chile	40	0	20	0
Uruguay	0	0	75	0
The Bahamas	25	100	25	25
Finland	14.25	20.55	66.3	14.25
Sweden	3.96	6.93	65.35	3.96
Norway	16.13	24.19	45.16	14.52
Mexico	21.67	55	36.67	21.67
Cuba	37.5	75	62.5	37.5
Dominican Republic	40	40	60	40
Belize	33.33	100	33.33	33.33
Jamaica	100	0	0	0
Nicaragua	20	60	40	20
Colombia	37.5	59.38	50	37.5
Venezuela	50	50	87.5	50
Netherlands	0	100	0	0
Kazakhstan	48	76	58.67	46.67
Ukraine	28.85	67.31	51.92	28.85
China	37.87	68.56	34.65	28.96
Mongolia	33.33	33.33	50	16.67
Switzerland	50	50	50	50
Hungary	16.67	66.67	16.67	16.67
Republic of Serbia	33.33	66.67	33.33	33.33
Estonia	0	0	100	0
Italy	15.38	65.38	19.23	15.38
Albania	28.57	85.71	42.86	28.57

Latvia	0	0	100	0
Turkey	45.45	81.82	47.73	45.45
Japan	30	70	50	30
Iraq	60	90	70	60
Syria	0	75	0	0
Romania	54.55	81.82	59.09	54.55
France	30	50	35	30
Poland	50	100	50	50
India	53.79	94.48	55.17	52.41
Slovakia	100	100	100	100
Pakistan	16.67	55.56	27.78	11.11
Moldova	80	100	80	80
Australia	15.62	31.25	53.12	15.62
Spain	17.07	56.1	19.51	17.07
Bulgaria	20	80	20	20
Georgia	0	0	33.33	0
Macedonia	100	100	100	100
Greece	16.67	61.11	16.67	16.67
Iceland	83.33	100	83.33	83.33
Portugal	33.33	66.67	16.67	16.67
Algeria	12.5	75	12.5	12.5
Tunisia	100	100	100	100
Morocco	33.33	77.78	33.33	33.33
Myanmar	0	33.33	33.33	0
Afghanistan	0	100	0	0
Thailand	71.43	78.57	78.57	71.43
Egypt	50	75	50	50
Cambodia	33.33	50	50	33.33
Mali	50	100	50	50
Iran	50	100	50	50
Vietnam	16.67	83.33	16.67	16.67
Bangladesh	28.57	85.71	28.57	28.57
Cameroon	0	100	0	0
Saudi Arabia	50	50	100	50
Ghana	66.67	66.67	100	66.67
Philippines	50	50	100	50
Sri Lanka	72.73	100	72.73	72.73
Malaysia	0	0	0	0
Uganda	0	100	0	0
Kenya	50	100	50	50
Burundi	0	0	0	0
Denmark	0	0	0	0

United Republic of Tanzania	16.67	16.67	66.67	16.67
Zambia	16.67	33.33	33.33	0
Democratic Republic of the Congo	60	80	80	60
United Kingdom	0	50	0	0
Madagascar	16.67	41.67	41.67	16.67
Zimbabwe	0	50	0	0
Mozambique	10	60	20	10
Botswana	0	0	100	0
South Africa	11.76	52.94	23.53	11.76
Germany	100	100	100	100
Czech Republic	0	100	0	0
Slovenia	0	0	100	0
Bosnia and Herzegovina	50	50	100	50
Montenegro	0	100	0	0
North Korea	75	75	100	75
South Korea	100	100	100	100
Taiwan	100	100	100	100
Laos	0	100	0	0
Burkina Faso	0	0	0	0
Swaziland	100	100	100	100
Haiti	0	0	100	0
El Salvador	0	100	0	0

Supplementary Table 6 | Percentage of studied lakes within a continent that experienced an increase in the occurrence frequency of bivariate and multivariate extremes.

	**Bivariate**			**Multivariate**
**Continent**	**Change in Bloom Occurrence and Low Water**	**Change in Bloom Occurrence and Heatwave**	**Change in Heatwave and Low Water**	**Change in all three extremes**
Europe	20.48	36.92	54.9	20.1
South America	24.44	35.56	47.41	22.59
North America	20.56	52.3	33.53	19.16
Asia	42.46	75.06	43.86	37.21
Oceania	15.62	31.25	53.12	15.62
Africa	26.26	59.6	39.39	25.25

Supplementary Table 7 | List of lakes that were analysed to validate the surface area time series.

HydroLake ID	Lake Name	Country	Longitude	Latitude	Correlation	Data source
5	Superior	USA	-84.461	46.469	0.31	DAHITI
8	Huron	USA	-82.423	42.999	0.86	DAHITI
9	Erie	USA	-78.908	42.904	0.4	DAHITI
16	Victoria	Uganda	33.194	0.431	0.5	DAHITI
17	Tanganyika	Democratic Republic of the Congo	29.185	-5.911	0.41	DAHITI
18	Malawi	Malawi	35.236	-14.418	0.61	DAHITI
53	Manitoba	Canada	-98.729	51.588	0.83	DAHITI
61	Red Lake Reservoir	USA	-95.272	47.956	0.4	USACE
65	Oahe	USA	-100.398	44.456	0.97	DAHITI
67	Great Salt	USA	-112.831	41.41	0.65	DAHITI
69	Okeechobee	USA	-81.101	26.941	0.4	DAHITI
70	Chapala	Mexico	-102.794	20.314	0.79	DAHITI
71	Managua	Nicaragua	-86.107	12.203	0.9	DAHITI
72	Nicaragua	Nicaragua	-84.782	11.122	0.4	DAHITI
73	Guri Reservoir	Venezuela	-62.998	7.764	0.93	DAHITI
76	Tucurui Reservoir	Brazil	-49.647	-3.833	0.86	DAHITI
77	Sobradinho Reservoir	Brazil	-40.824	-9.423	0.92	DAHITI
80	Ilha Solteira Reservoir	Brazil	-51.377	-20.373	0.66	DAHITI
115	Zeyskoye Reservoir	Russia	127.307	53.771	0.67	DAHITI
122	Zaysan	Kazakhstan	83.348	49.656	0.77	DAHITI
128	Tsimlyanskoye Reservoir	Russia	42.11	47.61	0.72	DAHITI
151	Poyang	China	116.221	29.752	0.65	DAHITI
152	Nasser	Egypt	32.886	23.967	0.95	DAHITI
156	Volta	Ghana	0.06	6.303	0.96	DAHITI
159	Albert	Uganda	31.41	2.761	0.62	DAHITI
162	Edward	Democratic Republic of the Congo	29.602	-0.141	0.63	DAHITI
163	Kivu	Rwanda	28.893	-2.489	0.51	DAHITI
169	Bangweulu	Zambia	29.815	-11.431	0.51	DAHITI
171	Cahora Bassa Reservoir	Mozambique	32.704	-15.586	0.93	DAHITI
172	Kariba Reservoir	Zambia	28.76	-16.523	0.98	DAHITI
715	Lake Koocanusa	USA	-115.314	48.411	0.72	USACE
719	Pend Oreille Lake	USA	-116.998	48.178	0.54	USACE
721	Fort Peck	USA	-106.415	48.001	0.98	DAHITI

730	Flathead Lake	USA	-114.232	47.676	0.66	DAHITI
796	Harry S. Truman Reservoir	USA	-93.406	38.265	0.68	USACE
804	Table Rock Lake	USA	-93.312	36.598	0.44	USACE
805	John H. Kerr Reservoir	USA	-78.295	36.596	0.47	USACE
807	Oologah Lake	USA	-95.679	36.424	0.62	USACE
809	Mead	USA	-114.735	36.018	1	DAHITI
811	Greers Ferry Lake	USA	-91.993	35.521	0.66	USACE
815	Eufaula Lake	USA	-95.358	35.308	0.7	USACE
821	Sardis Lake	USA	-89.793	34.407	0.76	USACE
823	Lake Sidney Lanier	USA	-84.072	34.161	0.71	USACE
825	Lake Texoma	USA	-96.573	33.823	0.73	USACE
826	Grenada Lake	USA	-89.769	33.815	0.77	USACE
832	Lake Tawakoni	USA	-95.915	32.815	0.82	DAHITI
836	Richland-Chambers Reservoir	USA	-96.103	31.948	0.91	DAHITI
837	Eufaula	USA	-85.065	31.628	0.4	USACE
839	Sam Rayburn Reservoir	USA	-94.106	31.065	0.83	USACE
1251	Balaton	Hungary	18.045	46.912	0.48	DAHITI
1348	Ataturk Dam	Turkey	38.323	37.485	0.92	DAHITI
1365	Assad	Syria	38.559	35.855	0.78	DAHITI
1423	Beas	India	75.949	31.973	0.93	India
1484	Gandhisagar Reservoir	India	75.554	24.7	0.97	India
1504	Ukal	India	73.598	21.256	0.86	DAHITI
1519	Nagarjuna	India	79.31	16.573	0.93	India
1521		India	75.888	16.333	0.9	India
1543	Roseires Reservoir	Sudan	34.39	11.798	0.86	DAHITI
1626	Chiuta	Mozambique	35.934	-14.349	0.57	DAHITI
1632	Argyle Reservoir	Australia	128.741	-16.118	0.96	BOM
1640	Dalrymple	Australia	147.138	-20.644	0.91	BOM
1650	Fairbairn	Australia	148.066	-23.653	0.98	BOM
1660	Wivenhoe	Australia	152.605	-27.392	0.99	BOM
8878	Marsh Lake	USA	-96.091	45.174	0.42	USACE
9104	Lost Creek Lake	USA	-122.672	42.673	0.9	USACE
9146	Coralville Lake	USA	-91.531	41.728	0.49	USACE
9157	Lake Red Rock	USA	-92.982	41.37	0.7	USACE
9160	Mosquito Creek Lake	USA	-80.756	41.302	0.4	USACE
9167	Berlin Lake	USA	-81.005	41.043	0.55	USACE
9169	Rathbun Lake	USA	-92.881	40.832	0.78	USACE
9176	Mississinewa Lake	USA	-85.955	40.714	0.46	USACE
9197	Harlan County Lake	USA	-99.215	40.073	0.94	USACE

9209	Mark Twain Lake	USA	-91.648	39.526	0.65	USACE
9217	Lake Shelbyville	USA	-88.777	39.411	0.52	USACE
9224	Tuttle Creek Lake	USA	-96.594	39.26	0.69	USACE
9226	Perry Lake	USA	-95.432	39.112	0.7	USACE
9228	Milford Lake	USA	-96.9	39.078	0.76	USACE
9229	Monroe Lake	USA	-86.5	39.008	0.43	USACE
9231	Wilson Lake	USA	-98.494	38.964	0.72	USACE
9232	Clinton Lake	USA	-95.331	38.919	0.63	USACE
9237	Hillsdale Lake	USA	-94.903	38.657	0.83	USACE
9238	Pomona Lake	USA	-95.557	38.653	0.67	USACE
9240	Kanopolis Lake	USA	-97.966	38.609	0.76	USACE
9242	Melvern Lake	USA	-95.716	38.514	0.67	USACE
9248	Marion Lake	USA	-97.083	38.371	0.78	USACE
9254	John Redmond Lake	USA	-95.765	38.24	0.71	USACE
9262	John Martin Reservoir	USA	-102.94	38.065	0.99	USACE
9264	Rend Lake	USA	-88.969	38.037	0.56	USACE
9269	El Dorado Lake	USA	-96.814	37.841	0.74	USACE
9296	Copan Lake	USA	-95.973	36.886	0.64	USACE
9299	Great Salt Plains Lake	USA	-98.14	36.747	0.55	USACE
9300	Kaw Lake	USA	-96.927	36.702	0.81	USACE
9323	Abiquiu Reservoir	USA	-106.429	36.238	0.97	USACE
9328	Keystone Lake	USA	-96.256	36.152	0.73	USACE
9331	Canton Lake	USA	-98.602	36.086	0.9	USACE
9333	Falls Lake	USA	-78.582	35.945	0.67	USACE
9344	B. Everett Jordan Lake	USA	-79.069	35.657	0.54	USACE
9348	Tenkiller Lake	USA	-95.037	35.595	0.57	USACE
9354	Conchas Lake	USA	-104.191	35.401	0.99	USACE
9364	Blue Mountain Lake	USA	-93.651	35.103	0.53	USACE
9365	Santa Rosa Lake	USA	-104.688	35.029	0.97	USACE
9371	Wister Lake	USA	-94.719	34.94	0.4	USACE
9378	Arkabutla Lake	USA	-90.123	34.756	0.66	USACE
9406	Broken Bow Lake	USA	-94.684	34.148	0.58	USACE
9411	Hugo Lake	USA	-95.383	34.011	0.69	USACE
9414	Pat Mayse Lake	USA	-95.557	33.854	0.76	USACE
9420	Millwood Lake	USA	-93.966	33.695	0.41	USACE
9430	Ray Roberts Lake	USA	-97.052	33.356	0.97	USACE
9434	Texarkana Lake	USA	-94.162	33.306	0.48	USACE
9443	Lewisville Lake	USA	-96.97	33.07	0.88	USACE
9446	Lavon Lake	USA	-96.47	33.033	0.9	USACE
9450		USA	-97.058	32.973	0.86	USACE
9462	Lake of the Pines	USA	-94.506	32.752	0.6	USACE

9465	Caddo Lake	USA	-93.916	32.707	0.58	USACE
9467		USA	-97.455	32.651	0.81	USACE
9474	Okatibbee Lake	USA	-88.792	32.475	0.52	USACE
9482		USA	-96.648	32.253	0.68	USACE
9487		USA	-96.699	31.953	0.69	USACE
9492	Lake Whitney	USA	-97.37	31.87	0.84	USACE
9496		USA	-97.198	31.577	0.82	USACE
9507		USA	-97.474	31.11	0.75	USACE
9508		USA	-97.533	31.023	0.81	USACE
9513	B.A. Steinhagen Lake	USA	-94.174	30.8	0.8	USACE
9517	Laneport Reservoir	USA	-97.337	30.69	0.73	USACE
9528		USA	-96.524	30.315	0.8	USACE
9542		USA	-98.2	29.872	0.72	USACE
15304		India	78.476	30.375	0.89	India
15600		India	79.224	21.662	0.9	India
15625		India	74.714	20.477	0.93	India
15760		India	76.489	13.886	0.96	India
16242		Australia	146.74	-19.412	0.97	BOM
16301		Australia	151.311	-24.071	0.94	BOM
16380	Somerset	Australia	152.557	-27.114	0.98	BOM
16414		Australia	151.219	-28.444	0.93	BOM
16473		Australia	150.928	-29.905	0.89	BOM
16508	Keepit	Australia	150.493	-30.878	0.96	BOM
16573	Burrendong	Australia	149.11	-32.669	0.96	BOM
16611	Wyangala	Australia	148.952	-33.973	0.98	BOM
16636	Eildon	Australia	145.927	-37.219	0.93	BOM
16644		Australia	146.798	-37.906	0.87	BOM
108146	Bowman-Haley Lake	USA	-103.249	45.981	0.8	USACE
109208	Highway 75 Dam Reservoir	USA	-96.291	45.239	0.53	USACE
111863	Michael J. Kirwan Reservoir	USA	-81.079	41.154	0.41	USACE
111913	Branched Oak Lake	USA	-96.854	40.971	0.86	USACE
112223	Long Branch Lake	USA	-92.517	39.753	0.58	USACE
112241	Cherry Creek Lake	USA	-104.86	39.647	0.62	USACE
112265	Chatfield Lake	USA	-105.056	39.555	0.8	USACE
112352	Lake Mendocino	USA	-123.181	39.197	0.88	USACE
112406	Longview Lake	USA	-94.466	38.922	0.81	USACE
112604	Toronto Lake	USA	-95.922	37.742	0.53	USACE
112614	Fall River Lake	USA	-96.069	37.649	0.64	USACE

112778	Fort Supply Lake	USA	-99.566	36.551	0.46	USACE
113697		USA	-98.478	31.974	0.81	USACE
113709		USA	-97.207	31.9	0.86	USACE
182850		Australia	148.389	-21.137	0.95	BOM

Supplementary Table 8 | List of lakes that were analysed to validate the lake heatwave frequencies.

HydroLake ID	Lake Name	Country	Lake area (km^2)	Longitude	Latitude	Correlation	Source
7	Ontario	USA	19347	-77.518	43.585	0.91	NDBC (National Data Buoy Center, https://www.ndbc.noaa.gov/)
66	Saint Clair	Canada	1161	-82.747	42.415	0.96	NDBC (National Data Buoy Center, https://www.ndbc.noaa.gov/)
104	Vattern	Sweden	1888	14.45	58.238	0.79	Woolway et al., (2019)
809	Mead	USA	581	- 114.407	36.15	0.95	NDBC (National Data Buoy Center, https://www.ndbc.noaa.gov/)
1242	Neusiedler See	Hungary	142	16.781	47.87	0.52	Dr. Martin Dokulil
8476	Sammamish	USA	19	- 122.096	47.603	0.81	King County government (https://green2.kingcounty.gov/)
8478	Washingon	USA	84	- 122.261	47.611	0.92	King County government (https://green2.kingcounty.gov/)
8736	Trout	USA	15	-89.668	46.031	0.61	NTL LTER (North Temperate Lakes LongTerm Ecological Research Network, https://lter.limnology.wisc.edu/)
9086	Mendota	USA	41	-89.42	43.111	0.66	NTL LTER (North Temperate Lakes LongTerm Ecological Research Network, https://lter.limnology.wisc.edu/)
12809	Erken	Sweden	23	18.578	59.845	0.91	SITES (Swedish Infrastructure for Ecosystem Science) (https://data.fieldsites.se/)
13387	Windermere	United Kingdom	13	-2.935	54.379	0.95	NERC Environmental Information Data Centre (https://eip.ceh.ac.uk/data)
14061	Woerther See	Austria	19	14.229	46.619	0.78	Dr. Martin Dokulil
16649		New Zealand	80	176.249	-38.07	0.48	EDP (Environmental Data Portal, https://envdata.boprc.govt.nz/)
16654	Tarawera	New Zealand	41	176.42	- 38.201	0.59	EDP (Environmental Data Portal, https://envdata.boprc.govt.nz/)
162733	Bassenthwaite	United Kingdom	5	-3.207	54.643	0.69	Government of Ireland (https://data.gov.ie/)
163604	Lough Feeagh	Ireland	4	-9.576	53.951	0.85	Government of Ireland (https://data.gov.ie/)
184967	Rotoehu	New Zealand	7	176.531	- 38.024	0.49	EDP (Environmental Data Portal, https://envdata.boprc.govt.nz/)

Supplementary References

1. Kottek, M., Grieser, J., Beck, C. et al. World map of the Köppen-Geiger climate classification updated. *Meteorol Z* **15**, 259–263 (2006).
