# Supplementary material for: Multivariate extremes in lakes
Source: Nat Commun. 2024 May 29;15:4559. doi: 10.1038/s41467-024-49012-7 (PMC11137041; doi:10.1038/s41467-024-49012-7)
Supplement: Supplementary file 3 — Description of Additional Supplementary Files [file 41467_2024_49012_MOESM3_ESM.pdf]

Description of Additional Supplementary Files

File Name: Supplementary Data 1
Description: General description of the 2,724 lakes included in this investigation. We show the location (longitude and latitude, country, continent) of each studied lake as well as the change in the occurrence frequency (%) of algal blooms, low water extremes and lake heatwaves. We also provide the HydroLake ID for each lake.
